# Atrial fibrillation detection by heart rate variability in Poincare plot

**DOI:** 10.1186/1475-925X-8-38

**Published:** 2009-12-11

**Authors:** Jinho Park, Sangwook Lee, Moongu Jeon

**Affiliations:** 1 Department of Information and Communications, Gwangju Institute of Science and Technology, 1 Oryong-dong, Buk-gu, Gwangju, Republic of Korea; 2 School of Information and Communication Engineering, Mokwon University, Mokwon Street 21, Doan-dong, Seo-gu, Deajon, Republic of Korea

## Abstract

**Background:**

Atrial fibrillation (AFib) is one of the prominent causes of stroke, and its risk increases with age. We need to detect AFib correctly as early as possible to avoid medical disaster because it is likely to proceed into a more serious form in short time. If we can make a portable AFib monitoring system, it will be helpful to many old people because we cannot predict when a patient will have a spasm of AFib.

**Methods:**

We analyzed heart beat variability from inter-beat intervals obtained by a wavelet-based detector. We made a Poincare plot using the inter-beat intervals. By analyzing the plot, we extracted three feature measures characterizing AFib and non-AFib: the number of clusters, mean stepping increment of inter-beat intervals, and dispersion of the points around a diagonal line in the plot. We divided distribution of the number of clusters into two and calculated mean value of the lower part by k-means clustering method. We classified data whose number of clusters is more than one and less than this mean value as non-AFib data. In the other case, we tried to discriminate AFib from non-AFib using support vector machine with the other feature measures: the mean stepping increment and dispersion of the points in the Poincare plot.

**Results:**

We found that Poincare plot from non-AFib data showed some pattern, while the plot from AFib data showed irregularly irregular shape. In case of non-AFib data, the definite pattern in the plot manifested itself with some limited number of clusters or closely packed one cluster. In case of AFib data, the number of clusters in the plot was one or too many. We evaluated the accuracy using leave-one-out cross-validation. Mean sensitivity and mean specificity were 91.4% and 92.9% respectively.

**Conclusions:**

Because pulse beats of ventricles are less likely to be influenced by baseline wandering and noise, we used the inter-beat intervals to diagnose AFib. We visually displayed regularity of the inter-beat intervals by way of Poincare plot. We tried to design an automated algorithm which did not require any human intervention and any specific threshold, and could be installed in a portable AFib monitoring system.

## Background

There is a growing tendency that atrial fibrillation (AFib) related disease affects quality of life. Its risk increases with age [1]; in fact, AFib is one of the most common types of arrhythmia in clinical practice [2]. Blood circulation of AFib patients is not smooth; therefore, AFib patients feel dizzy and uncomfortable while they exercise. AFib can be one of the deadliest symptoms to patients with preexcitation; in this case, it often induces tachycardia of ventricles or atrioventricular fibrillation [3]. The more serious effect of AFib is formation of blood clots by congestion of blood in atria [2]. If these blood clots come out of the atria and occlude a vessel somewhere in the brain, dire stroke can come about.

AFib can be classified into three grades: paroxysmal, persistent and permanent AFib. The paroxysmal AFib can be a preceding omen of the persistent AFib. Takahashi, Seki and Imatak observed that their patients with the paroxysmal AFib were highly affected with the more serious form of AFib; 25.3% of paroxysmal AFib patients developed into the more serious form of AFib in one year [4].

Electrical remodeling is one of the features of AFib and it is related to decreased conduction velocity of electricity signals [2]. When the heart experiences the electrical remodeling, an area of slow conduction takes place in atria because of insufficient recovery of excitability. Slow conduction shortens wavelengths of the wandering electricity signals; thus, this area of slow conduction increases the number of re-current wave fronts of depolarization in atria and contributes to the sustaining of AFib [5]. The wandering wave fronts around the atria fork themselves or collide with one another; accordingly, these maintain the turbulence process of electric conduction in atria [6]. The re-entrant wave fronts induce inappropriate heart pumping; consequently, they deteriorate solidity of cardiac hemodynamics.

There are several studies about screening AFib by palpating an electrocardiogram (ECG) manually. Sudlow et al. tried to screen AFib by two methods: digoxin prescriptions and pulse palpation of ECG. Sensitivity and specificity using digoxin prescriptions were somewhat low. Sensitivity and specificity using pulse palpation were (93%, 71%) in case of women elder than 75, (100%, 86%) in case of 65-74 aged women, (95%, 71%) in case of men elder than 75, (100%, 79%) in case of 65-74 aged men, (sensitivity, specificity) respectively [7]. Somerville et al. reported a screening result of AFib; Sensitivity and specificity were 100% and 77% respectively [8]. Mant et al. showed the screening result by general practitioners and practice nurses observing 12 lead ECG. Sensitivity and specificity were 79.8% and 91.6% by general practitioners, 77.1% and 85.1% by practice nurses [9].

There are lots of studies about detecting AFib. Xu et al. chose five feature parameters which were input regularity, input atrial rate, energy distribution, time interval corresponding to zero amplitude signal, and number of points reaching baseline. They used Bayesian discriminator to classify the input data as one of sinus rhythm, AFib or atrial flutter [10]. Petrucci et al. used two histograms which were calculated from the inter-beat intervals. One histogram consisted of differences between two successive inter-beat intervals and the other histogram consisted of normalized deviations from mean value of the inter-beat intervals. They calculated distribution widths from these histograms to discriminate AFib from non-AFib [11]. Kikillus et al. made a Poincaré plot from inter-beat intervals and estimated density of points in each segment of Poincaré plot. They calculated an indicator of AFib from standard deviation of temporal differences of the consecutive inter-beat intervals [12]. Thuraisingham used wavelet method to obtain a filtered time series from the input ECG. He calculated the standard deviation of the time series and the standard deviation of successive differences, and the length of the ellipse that characterized the Poincaré plot. He used these indicators to discriminate AFib from non-AFib [13]. Shouldice et al. made feature vectors from inter-beat intervals, and then applied Fisher's linear discriminant method to estimate the likelihood of a block of inter-beat intervals containing the paroxysmal AFib [14]. Kikillus et al. tried to detect AFib using a method of neural network. They calculated 25 parameters of time domain, frequency and non-linear domain, with which they applied two neural networks to decide whether the input ECG implied AFib [15].

If the paroxysm of AFib occurs, variability of the inter-beat intervals increases from the onset to the end of AFib [16]; hence, we analyzed the pulse-beat patterns to detect AFib. If the input data is contaminated with noise, it's difficult to discriminate fibrillatory wave from the noise; on the other hand, the pulse beat patterns in ECG are less likely to be influenced by baseline wandering and noise because they have clear appearances. We tried to design an algorithm which could be installed in a portable heart monitoring system since we cannot predict when the paroxysm of AFib will come about. It should endure noise well to diagnose AFib in a mobile situation. In this regard, we focused on dynamics of the inter-beat intervals to detect the onset of AFib.

## Methods

### ECG data

We used two databases, Computers in Cardiology challenge 2001 and 2004 (CinC 2001, 2004) of physionet [17,18]. The CinC 2001 database includes both AFib and non-AFib data files. These files were made from 24 hour ECG by cutting appropriate segments, and came from 48 different people. The files whose names begin with 'n' contain the ECG data from people who do not have any AFib. However, those people had several diseases only except AFib or else they were normal. Even numbered files whose names begin with 'p' and end with 'c' contain AFib ECG data. We dissected each data into one minute quantity to analyze easily and took only first one minute amount. The CinC 2004 database includes only AFib data files. Each file of the database had one minute amount of data. Sampling frequency of each file was 128 Hz. Each ECG data has two simultaneous components which record two different leads of ECG. We chose the first component. ECG files 'n27' and 'n27c' from the CinC 2001 database had too much noise, so we could not detect heart beat well; hence, we omitted two files. We used 25 AFib and 98 non-AFib data files from the CinC 2001 database and 80 AFib data files from the CinC 2004 database. Almost two non-AFib data files were obtained from one person and only one AFib data file was obtained from each patient. There was no explanation about the number of patients in the CinC 2004 database.

### Inter-beat intervals

**Figure 1:**
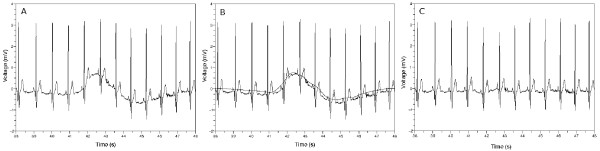
**Removing baseline wandering**. (a) Original waveform. The baseline of the waveform is fluctuating. (b) Waveform obtained by applying inverse wavelet transform to adequately chosen coefficient vectors. This waveform is overlapped with the waveform of the left figure. (c) Subtraction result. There is no baseline wandering. The details of the leftmost figure were preserved except the baseline.

We obtained inter-beat intervals from input ECG data by using the wavelet method [19]. We present an overview here. First we applied a discrete wavelet transform on an input ECG data to find transform coefficient vectors

where *A*_*N *_is an approximation coefficient vector, and *D*_*i*_, (*i *= 1,..., *N*) is a detail coefficient vector. We chose one detail coefficient vector *D*_*i *_by a criterion [19], and assigned zeros to the detail coefficient vectors *D*_*i*-1_, *D*_*i*-2_,⋯, *D*_1_. Figure 1(b) shows a waveform obtained by applying inverse wavelet transform to the coefficient vectors

where 0 means a zero vector. Figure 1(c) is a result obtained by subtracting the waveform of Figure 1(b) from the waveform of Figure 1(a). We can see the waveform of Figure 1(c) is leveled out and the details of the waveform were preserved with respect to the waveform of Figure 1(a).

Next we tried to find time position of each QRS complex which is protruded substantially above the baseline. The QRS complexes designate the heart beats. We calculated the approximation and detail coefficient vectors  by applying discrete wavelet transform to the waveform resulted from removing the baseline. Choosing one detail coefficient vector  we made new  by applying some treatments to the vector [19]. We assigned zero vectors to the other vectors and applied inverse wavelet transform to

**Figure 2:**
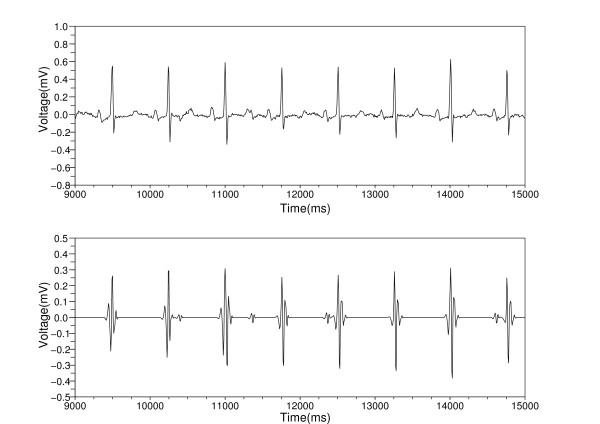
**Catching the time positions of the QRS complexes**. The top figure shows the waveform obtained by removing the baseline wandering. The bottom figure shows the result obtained by applying inverse wavelet transform. This waveform teaches us the time positions of the QRS complexes.

We determined the most adequate wavelet scale by comparing the Pearson correlation coefficients [19]. Figure 2 shows that the waveform obtained by inverse wavelet transform indicates the time positions of the QRS complexes.

### Poincaré plots

**Figure 3:**
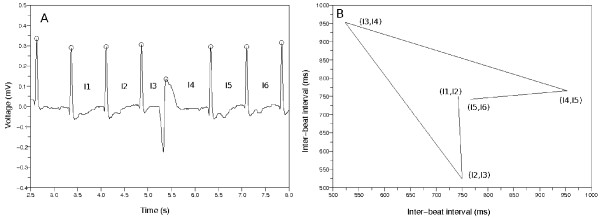
**The example of building a Poincaré plot**. (a) Sample ECG data. There is a premature ventricular contraction (PVC). The mark *O *means the detector catched the QRS complex. (b) Poincaré plot made from the inter-beat intervals {*I*_1_, *I*_2_, *I*_3_, *I*_4_, *I*_5_, *I*_6_}. We drew the lines between the consecutive points in this plot. The points make a wedge-shaped diagram because of the PVC.

If we represent the inter-beat intervals as a sequence *I*_1_, *I*_2_, *I*_3_, *I*_4_, *I*_5_,⋯, *I*_*n *_like Figure 3(a), we can make a Poincaré plot that is composed of the points (*I*_1_, *I*_2_), (*I*_2_, *I*_3_), (*I*_3_, *I*_4_), (*I*_4_, *I*_5_),⋯, (*I*_*n*-1_, *I*_*n*_). We connected the consecutive points with lines to observe dynamics of the inter-beat intervals.

The Poincaré plot applicable to discrete data is closely related to a conventional phase plane of continuous data. If an *x*-coordinate of a point in Poincaré plot is *x*_1_, *y*-coordinate of the point is mathematically related to [20]. If the *x *axis of phase plane is *x*, the *y *axis corresponds to .

Figure 3 describes the procedure of building a Poincaré plot. Figure 3(a) indicates an ECG data containing a premature ventricular contraction (PVC); in addition, it represents the inter-beat intervals *I*_1_, *I*_2_, *I*_3_, *I*_4_, *I*_5_, *I*_6_. Figure 3(b) describes the Poincaré plot made from these inter-beat intervals. This Poincaré plot has the points of (*I*_1_, *I*_2_), (*I*_2_, *I*_3_), (*I*_3_, *I*_4_), (*I*_4_, *I*_5_), (*I*_5_, *I*_6_), and we drew the lines between the consecutive points to observe the dynamics more easily. The points revolve clockwise and make a wedge-shaped diagram. This is because the inter-beat intervals changed around the PVC.

#### Typical patterns of Poincaré plots

**Figure 4:**
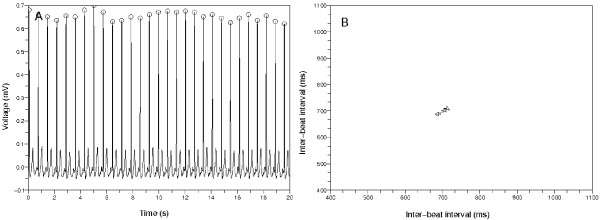
**First typical Poincaré plot from non-AFib ECG data**. (a) The 20 second amount of ECG data from 'n01', CinC 2001. The inter-beat intervals are uniform. The mark *O *means the QRS detector found the time position of the ventricular activity. (b) The Poincaré plot from the left ECG data. The points gather around one point on the diagonal line. This means the inter-beat intervals are almost same.

**Figure 5:**
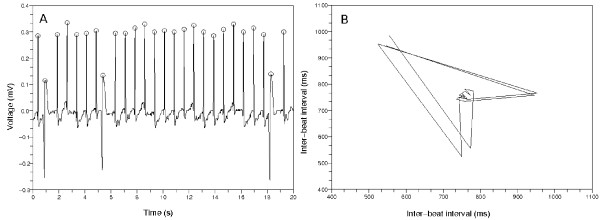
**Second typical Poincaré plot from a non-AFib ECG data**. (a) The 20 second amount of ECG data from 'n04', CinC 2001. There are PVCs. The inter-beat intervals change around the PVCs. (b) The points of this Poincaré plot revolve clockwise and make a wedge-shaped diagram. This is because the inter-beat intervals changed around the PVCs.

The Poincaré plots from non-AFib data show several typical patterns. Figure 4(a) represents an ECG data whose inter-beat intervals are uniformly distributed. The Poincaré plot in Figure 4(b) shows a pattern that the points congregate around one central point. This stands for the almost same inter-beat intervals between the former and the latter beats. The mark *O *means the QRS complex detector found the time position corresponding to the ventricular activity. Figure 5(a) shows some PVCs exist. The inter-beat intervals change around the PVCs. This is represented in the Figure 5(b) as a wedge-shaped Poincaré plot. This type of Poincaré plot is also reported in Zemaityte et al.'s paper [21]. The difference between the Poincaré plot in this paper and the plot in Zemaityte et al.'s paper is whether the lines are drawn or not between the consecutive points in the plots.

#### Poincaré plot in case of AFib

**Figure 6:**
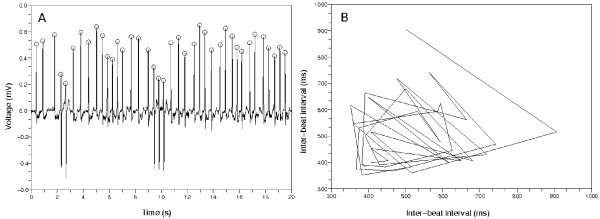
**Poincaré plot from an AFib ECG data**. (a) The 20 second amount of ECG data. The inter-beat intervals are irregularly distributed. The mark *O *means the QRS detector found the time position of the ventricular activity. (b) Their corresponding Poincaré plot. There is no pattern. The points in this plot often move across the diagonal line.

Figure 6 demonstrates that Poincaré plot does not have any specific pattern in case of AFib, and the points in the Poincaré plot move irregularly. This explains that the inter-beat intervals are statistically independent from each other under the state of AFib, except for a slight correlation between the immediate subsequent beats [20]. The points in the plot often move across the diagonal line. We drew the lines between the consecutive points in the Poincaré plot to observe movements of the points more easily. This plot is similar to many AFib plots in other papers [20,21].

### Feature selection

#### Mean stepping increment of inter-beat intervals

Let us assume that we were given inter-beat intervals, *I*_1_, *I*_2_, *I*_3_, *I*_4_, *I*_5_,⋯, *I*_*n*_. The points in Poincaré plot will be (*I*_1_, *I*_2_), (*I*_2_, *I*_3_),⋯, (*I*_*n*-1_, *I*_*n*_) in order. If we designate two consecutive points as (*I*_*j*_, *I*_*j*+1_) and (*I*_*j*+1_, *I*_*j*+2_), the distance between two points in the Poincaré plot will be . We calculated mean value of these quantities as . This implies rate of change of the inter-beat intervals in the Poincaré plot. To normalize this and make this quantity dimensionless, we divided it by mean inter-beat interval, . We defined next quantity as the mean stepping increment of the inter-beat intervals.

#### Dispersion of points around diagonal line in Poincaré plot

Let us calculate coordinates of a central point on the diagonal line in Poincaré plot. If the inter-beat intervals are *I*_1_, *I*_2_, *I*_3_, *I*_4_, *I*_5_,⋯, *I*_*n*_, the points of the Poincaré plot consist of (*I*_1_, *I*_2_), (*I*_2_, *I*_3_), (*I*_3_, *I*_4_),⋯, (*I*_*n*-1_, *I*_*n*_). We tried to find a central point (*x*, *x*) minimizing sum of distance squares from this point to all the other points in the Poincaré plot. If we designate this sum as *E*(*x*), this will be represented as follows.

To find the point minimizing this sum, we calculated a derivative with respect to the variable *x*. From , we found the central point (*a*, *a*) as follows.

The distance from a point (*I*_*j*_, *I*_*j*+1_) to the diagonal line *y *= *x *is represented as . Standard deviation of these terms is represented as follows.

This term can be used to indicate how spread the points in Poincaré plot are distributed around the diagonal line. We chose the following ratio of the above two terms as a distinguishing feature.

#### Number of clusters in Poincaré plot

To determine the number of clusters in Poincaré plot, we developed a clustering method based on spectral graph theory [22]. The Poincaré plots in Figure 4(b) and 5(b) show that non-AFib data sets have a limited number of clusters. On the other hand, Figure 6(b) shows that AFib data sets can have many clusters or just one conglomerate lump.

### Correcting faults in QRS complex detection

**Figure 7:**
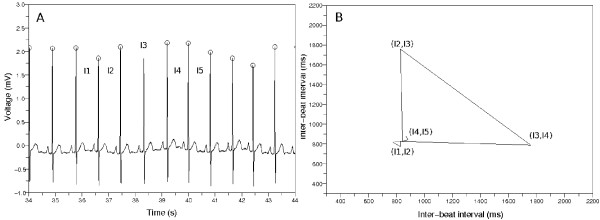
**Poincaré plot when the QRS detector misses one**. (a) The QRS detector missed one QRS complex. (b) Poincaré plot made from the wrong inter-beat intervals. We can find that the detector did not catch one QRS complex by investigating the Poincaré plot.

Poincaré plot helped us to catch a fault of our QRS complex detector. If the QRS complex detector misses one QRS complex like Figure 7(a), the inter-beat interval corresponding to that portion shall be longer than the other intervals. This is represented as a triangle in Figure 7(b) because the method of making a Poincaré plot produces the points (*I*_1_, *I*_2_), (*I*_2_, *I*_3_), (*I*_3_, *I*_4_), (*I*_4_, *I*_5_) where *I*_1_, *I*_2_, *I*_4 _and *I*_5 _are almost same but less than *I*_3_.

We tried to correct this by identifying the triangle in the Poincaré plot as follows. Let us designate two consecutive points as (*I*_*j*_, *I*_*j*+1_) and (*I*_*j*+1_, *I*_*j*+2_). We calculated the coordinates of the central point (*a*, *a*) on diagonal line in Poincaré plot in the above section. We can think of the central point (*a*, *a*) as a new origin; then the coordinates of two consecutive points will be (*I*_*j *_- *a*, *I*_*j*+1 _- *a*) and (*I*_*j*+1 _- *a*, *I*_*j*+2 _- *a*) with regard to this new origin. We identified a mistake done by the QRS complex detector when the distances , are similar and the coordinates of the middle point  are positive and this middle point is located near the diagonal line and the smaller of , is substantially large.

### Support vector machine classifier

We employed 1-norm support vector machine with radial basis function kernel. The forms of the support vector machine and the radial basis function kernel were given as follows.

The parameters *C *and *γ *were selected by an automatic tool provided by a support vector machine program [23]. We gave an option '-v 2' to this automatic tool.

## Results

We tried to detect AFib using the above three feature measures. First we extracted inter-beat intervals from an ECG data by the wavelet method [19] and made Poincaré plot using these inter-beat intervals. If there were a limited number of clusters in Poincaré plot like the Figure 5(b), we concluded the Poincaré plot implied non-AFib because the small plural number of clusters meant the ECG data had some pattern.

We know that the Poincaré plot of the Figure 4(b) has the points accumulated around a central point and the Poincaré plot of the Figure 6(b) has the points scattered all over the place. The Poincaré plot of the Figure 4(b) has one cluster, and the estimated number of clusters in the Figure 6(b) is one or too many. If there was an error of calculating the inter-beat intervals from AFib data, we often got the result that the number of clusters in the Poincaré plot was too many. We had to deal with this error situation.

**Figure 8:**
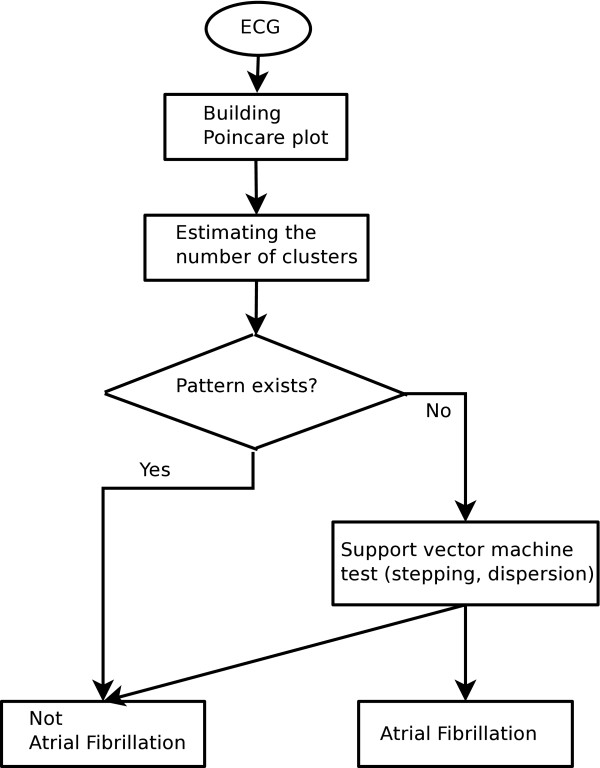
**Flow chart to discriminate AFib from non-AFib**. We tried to discriminate AFib from non-AFib using three feature measures. We divided the distribution of the number of clusters into two, and calculated the lower mean value by k-means clustering method. We decided there was a pattern in Poincaré plot if the number of clusters of a test data is more than one and less than this mean value. Otherwise we further applied support vector machine method to the other remaining features measures.

To remedy this problem, we used k-means clustering method to get a criterion about the number of clusters. We divided the distribution of the number of clusters into two, and calculated mean value of the lower part. If the number of clusters of a test data was larger than this mean value, we considered the inter-beat intervals of this test data had an error. In this way, we classified a test data as non-AFib if the number of clusters in the Poincaré plot was more than one and smaller than the above mean value obtained by k-means clustering method. In case that the number of clusters is one or too many with respect to the above criterion, we tried to discriminate AFib from non-AFib by way of support vector machine method using the other two feature measures: the mean stepping increment of inter-beat intervals, and dispersion of the points around a diagonal line. The whole process producing a detection result is depicted in Figure 8.

**Figure 9:**
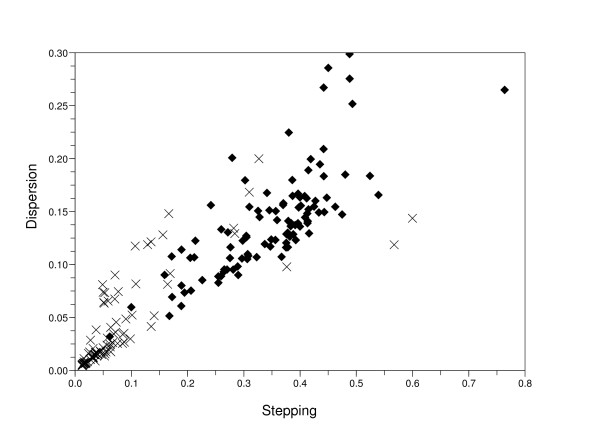
**A relation between two features, mean stepping increment of inter-beat intervals and dispersion of the points in Poincaré plot when we got one or too many number of clusters**. We used support vector machine method on the data whose number of clusters is one or too many to discriminate AFib from non-AFib. The mark *X *means non-AFib data and the diamond mark means AFib data.

We represent a plot in Figure 9 which shows a relation between two features, the mean stepping increment of inter-beat intervals and dispersion of the points in Poincaré plot in case that we had to apply support vector machine. This is when we got one or too many number of clusters. From this plot we can assume that the Figure 4(b) has relatively low dispersion and mean stepping increment; in contrast, the Figure 6(b) has high values.

We evaluated the accuracy using leave-one-out 4-fold cross-validation. That is, we divided the whole data into two groups which were test data occupying  amount out of total data and training data occupying  amount. We switched test data each time and took the remaining as a training data. We built a classifier using the training data each time, and did experiments with the test data by this classifier. Switching the test and training data four times, we tested sensitivity and specificity. True Positive in Table 1 indicates the number of data files which are AFib, and whose test outcomes are also AFib. True Negative indicates the number of data files which are non-AFib, and whose test outcomes are also non-AFib. False Positive indicates the number of data files which are non-AFib, but whose test outcomes are AFib. False Negative indicates the number of data files which are AFib, but whose test outcomes are non-AFib. Sensitivity and specificity were obtained as follows.

**Table 1:** The leave-one-out 4-fold cross-validation.

Test number	True Positive	True Negative	False Positive	False Negative	Sensitivity	Specificity
1	26	23	2	1	0.963	0.920

2	25	23	2	1	0.962	0.920

3	22	22	2	4	0.846	0.917

4	23	23	1	3	0.885	0.958

Mean sensitivity and mean specificity were obtained by Mean sensitivity = Total True Positive/(Total True Positive + Total False Negative), Mean specificity = Total True Negative/(Total False Positive + Total True Negative). Mean sensitivity and mean specificity were 91.4% and 92.9% respectively.

## Conclusions

We tried to propose an automated detection method which did not require any human intervention. Usually an ECG data is recorded for some period of time; then, an expert reads the data to find an abnormality in it. However, this process is tedious and inconvenient for patients since they should be in some place equipped with an electrocardiograph. Because pulse beats of ventricles are less likely to be influenced by baseline wandering and noise, we used the inter-beat intervals to diagnose AFib. It will be useful to make a real time portable monitoring electrocardiograph because we cannot predict when the paroxysm of AFib will come about. Our algorithm requires only one lead of ECG to acquire inter-beat intervals. We tried to design our algorithm not using any specific threshold.

Heart rate variability is closely related to homeostasis of the autonomous nervous system. The dynamics of inter-beat intervals come to change after the onset of AFib. Therefore we used the Poincaré plot because it was a useful tool in studying dynamics of ECG data. We found that people without any AFib showed some patterns in the Poincaré plots and these patterns were regular. The plots of AFib patients, however, were very irregular and changed too much in the course of time.

We described feature selection process and classifier using clustering and support vector machine method. We captured inter-beat intervals from an input ECG data, and drew Poincaré plot using them. By analyzing the Poincaré plot, we obtained three feature measures: the number of clusters, mean stepping increment of inter-beat intervals, and dispersion of the points in the plot. These three feature measures were dimensionless quantities.

There are some limitations in this paper. First, one feature, the dispersion of points in Poincaré plot, was not powerful to discriminate AFib from non-AFib as you can see in the Figure 9. Second, the number of data used in this paper was somewhat small. Third, the approach taken by us is susceptible to performance of QRS complex detector. If the QRS complex detector made a mistake and we failed to correct it, the accuracy of this algorithm would greatly fall off.

## Competing interests

The authors declare that they have no competing interests.

## Authors' contributions

JP has worked on the wavelet analysis, extracted three features by investigating the Poincaré plots, and written the whole manuscript. SL has worked on classification using the support vector machine. MJ has designed the detection strategy of AFib, and corrected the manuscript. All authors have read and approved the final manuscript.
